# Information decomposition in complex systems via machine learning

**DOI:** 10.1073/pnas.2312988121

**Published:** 2024-03-18

**Authors:** Kieran A. Murphy, Dani S. Bassett

**Affiliations:** ^a^Department of Bioengineering, School of Engineering & Applied Science, University of Pennsylvania, Philadelphia, PA 19104; ^b^Department of Electrical & Systems Engineering, School of Engineering & Applied Science, University of Pennsylvania, Philadelphia, PA 19104; ^c^Department of Neurology, Perelman School of Medicine, University of Pennsylvania, Philadelphia, PA 19104; ^d^Department of Psychiatry, Perelman School of Medicine, University of Pennsylvania, Philadelphia, PA 19104; ^e^Department of Physics & Astronomy, College of Arts & Sciences, University of Pennsylvania, Philadelphia, PA 19104; ^f^The Santa Fe Institute, Santa Fe, NM 87501

**Keywords:** information theory, machine learning for science, complex systems, amorphous plasticity

## Abstract

One of the fundamental steps toward understanding a complex system is identifying variation at the scale of the system’s components that is most relevant to behavior on a macroscopic scale. Mutual information provides a natural means of linking variation across scales of a system due to its independence of functional relationship between observables. However, characterizing the manner in which information is distributed across a set of observables is computationally challenging and generally infeasible beyond a handful of measurements. Here, we propose a practical and general methodology that uses machine learning to decompose the information contained in a set of measurements by jointly optimizing a lossy compression of each measurement. Guided by the distributed information bottleneck as a learning objective, the information decomposition identifies the variation in the measurements of the system state most relevant to specified macroscale behavior. We focus our analysis on two paradigmatic complex systems: a Boolean circuit and an amorphous material undergoing plastic deformation. In both examples, the large amount of entropy of the system state is decomposed, bit by bit, in terms of what is most related to macroscale behavior. The identification of meaningful variation in data, with the full generality brought by information theory, is made practical for studying the connection between micro- and macroscale structure in complex systems.

## Significance

A defining characteristic of complex systems is an abundance of variation at one scale of observation that contains, hidden within, information about organization at another scale. To see the forest through the trees is a challenge faced whether studying society or a sandpile, climate, or a brain. We present a fully general and practical methodology, rigorously grounded in information theory, that surfaces important information out of a sea of variation in a comprehensible manner. At its core is the concept of lossy compression: Some information in a measurement is preserved, and the rest is discarded. We use machine learning to lossily compress tens to hundreds of measurements simultaneously, providing a route to insight about complex systems through information decomposition.

A complex system is a system of interacting components where some sense of order present at the scale of the system is not apparent, or even conceivable, from the observations of single components (1, 2). A broad categorization, it includes many systems of relevance to our daily lives, from the economy to the internet and from the human brain to artificial neural networks (3, 4). Before attempting a reductionist description of a complex system, one must first identify variation in the system that is most relevant to emergent order at larger scales. The notion of relevance can be formalized with information theory, wherein mutual information serves as a general measure of statistical dependence to connect variation across different scales of system behavior (5, 6). Information theory and complexity science have a rich history; information theory commonly forms the foundation of definitions of what it means to be complex (7–11).

Machine learning is well suited for the analysis of complex systems, grounded in its natural capacity to identify patterns in high dimensional data (12). However, distilling insight from a successfully trained model is often infeasible due to a characteristic lack of interpretability of machine learning models (13, 14). Restricting to simpler classes of models, for example linear combinations of observables, recovers a degree of interpretability at the expense of functional expressivity (15). For the study of complex systems, such a trade-off is unacceptable if the complexity of the system is no longer faithfully represented. In this work, we do not attempt to explain the relationship between microscale and macroscale and are instead interested in identifying the information contained in microscale observables that is most predictive of macroscale behavior—independent of functional relationship.

We employ a recent method from interpretable machine learning that identifies the most relevant information in a set of measurements (16). Based on the distributed information bottleneck (17, 18), a variant of the information bottleneck (IB) (19), the method lossily compresses a set of measurements while preserving information about a relevance quantity. Optimization serves to decompose the information present in the measurements, providing a general-purpose method to identify the important variation in composite measurements of complex systems.

Identifying important variation is a powerful means of analysis of complex systems, as we demonstrate on two paradigmatic examples. First, we study a Boolean circuit, whose fully specified joint distribution and intuitive interactions between variables facilitate understanding of the information decomposition found by the distributed IB. Boolean circuits are networks of binary variables that interact through logic functions, serving as the building blocks of computation (20) and as elementary models of gene control networks (21, 22). Second, we decompose the information contained in the fine-grained positional configuration of an amorphous material subjected to global deformation. Amorphous materials are condensed matter systems composed of simple elements (e.g., atoms or grains) whose positional disorder gives rise to a host of complex macroscale phenomena, such as collective rearrangement events spanning a wide range of magnitudes (23, 24) and nontrivial phase transitions (25–28). Although the state space that describes all of the degrees of freedom is large, as is generally true of complex systems, the proposed method is able to identify the most important bits of variation by leveraging machine learning to navigate the high-dimensional space of possible lossy compression schemes.

## 1. Methods

Mutual information is a measure of statistical dependence between two random variables *X* and *Y* that is independent of the functional transformation that relates *X* and *Y* (in contrast to linear correlation, for example, which measures the degree to which two variables are linearly related). Mutual information is defined as the entropy reduction in one variable after observing the value of the other variable (29),

[1] $$\begin{array}{c} I(X;Y)=H(Y)-H(Y|X), \end{array}$$

with $H\left(X\right)=E_{x∼p(x)}\left[-log\;p\left(x\right)\right]$ Shannon’s entropy (30).

The IB (19) is an optimization objective to extract information contained in a variable *X* that is most relevant to another variable *Y*. Under the IB, *X* is lossily compressed to an auxiliary random variable U=f(X) that simultaneously maximizes I(U;Y) and minimizes I(U;X). Consider the case where *X* is a composite measurement—e.g., multiple degrees of freedom of a complex system—more appropriately denoted as a random vector $X=(X_{1},...,X_{N})$. By optimizing the IB, a lossy compression *U* of X can be found that extracts information relevant to *Y*, but without any indication from which components the information originated. If, instead, an IB is installed on each of the components, each $X_{i}$ undergoes lossy compression to a corresponding $U_{i}$, and then the elements of the vector $U=(U_{1},...,U_{N})$ contain the relevant information extracted from each component. Known as the distributed IB (17, 31), configuring IBs in this way enables a detailed analysis of the structure of information with respect to the components (16). Minimization of the distributed IB Lagrangian,

[2] $$\begin{array}{c} L_{DIB}=\beta \sum_{i=1}^{N}I(U_{i};X_{i})-I(U;Y), \end{array}$$

extracts the entropy in X that is most descriptive of *Y*. By sweeping over the magnitude of the bottleneck strength *β*, a continuous spectrum of approximations to the relationship between X and *Y* is found, each utilizing a different amount of information from X. The product of optimization is thus a sequence of distributed compression schemes, U(β), more appropriately parameterized by the total utilized information $\sum_{i=1}^{N}I\left(U_{i};X_{i}\right)$. The central focus of this work is to connect the optimized U to the structure of information contained in X for insight about the complex system under study.

In place of Eq. **2**, variational bounds on mutual information have been developed that are amenable to data and machine learning (18, 32). The lossy compression schemes are parameterized by neural networks that encode data points to probability distributions in a continuous latent space. Stochasticity in the mapping between $X_{i}$ and $U_{i}$ allows the transmitted information to be smoothly varied and consequently facilitates optimization. The amount of transmitted information is upper bounded by the expectation of the Kullback–Leibler divergence (29) between the encoded distributions and an arbitrary prior distribution, identical to the process of information restriction in a variational autoencoder (32, 33). While training utilizes the aforementioned upper bound, for evaluation over the course of an optimization, we desire more accurate estimates of the information $I(U_{i};X_{i})$ extracted from each component. We estimated upper and lower bounds derived in ref. 34 to obtain precise estimates of each mutual information term, where the interval between bounds was on the order of 0.01 bits. Although mutual information is generally difficult to estimate from data (35), compressing the partial measurements $X_{i}$ separately segregates the information such that the amount of mutual information is small enough to allow precise estimates. Characterization of the mutual information estimation may be found in *SI Appendix*.

## 2. Results

### 2.1. Boolean Circuit.

**Fig. 1.**
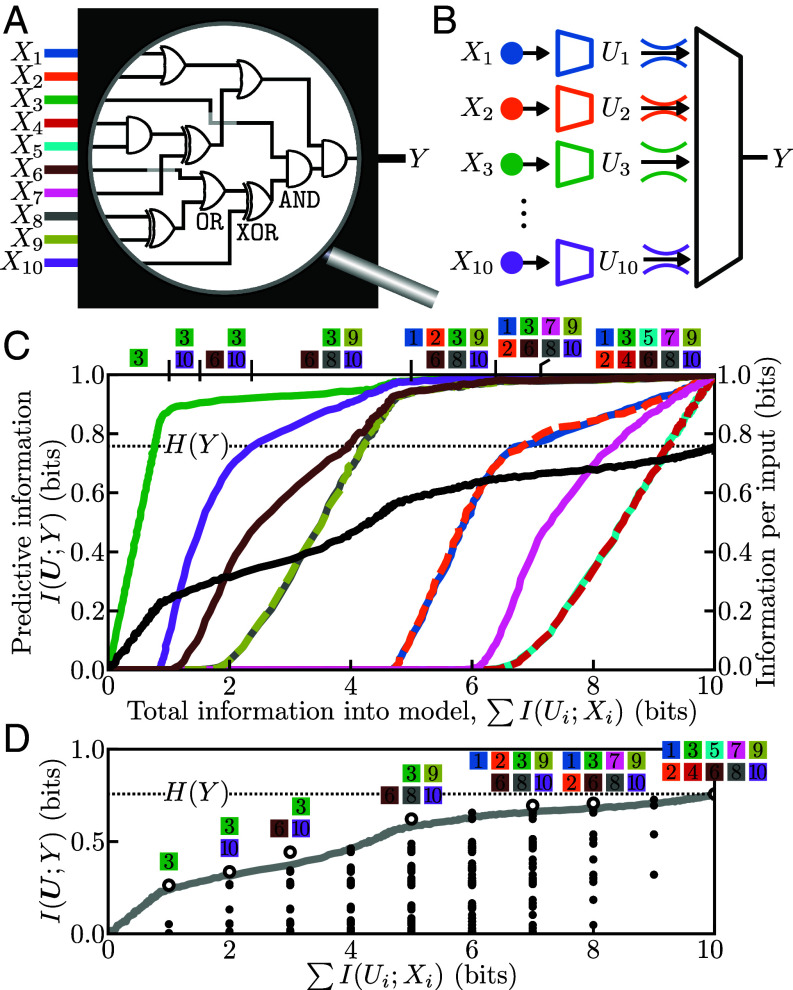
Decomposing the information contained in the inputs of a Boolean circuit. (*A*) Ten binary inputs $X=(X_{1},...,X_{10})$ are connected via AND, OR, and XOR gates to a binary output *Y*. (*B*) A lossy compression $U_{i}$ is learned for each $X_{i}$, and then all $U_{i}$ are combined as input to a machine learning model trained to predict *Y*. (*C*) The distributed information plane displays the predictive information about the output (left vertical axis, black) as a function of the total information utilized about the input. For each value of total information into the model, there is an allocation of information to the input gates indicating their relevance to the output *Y* [right vertical axis, colors corresponding to input gates in panel (*A*)]. The subset of inputs identified as containing the most relevant information ($I(U_{i};X_{i})\geq 0.1$ bits) is indicated at the top of the plot. Dashed lines are used for the information allocations when there is significant overlap. (*D*) The mutual information between all subsets of input channels and *Y* are displayed on the distributed information plane as black circles. The optimization of the distributed IB (gray curve) identified subsets of inputs that contain the most predictive information (open circles).

A randomly generated Boolean circuit with ten binary inputs $X=(X_{1},...,X_{10})$ and a binary output *Y* is shown in Fig. 1*A*. Assuming a uniform distribution over inputs, the truth table specifies the joint distribution $p(x_{1},...,x_{10},y)$, and the interactions between inputs are prescribed by a wiring of logical AND, OR, and XOR gates. An IB was distributed to every input $X_{i}$ to monitor from where the predictive information originated via compressed variables $U_{i}$ (Fig. 1*B*). We trained a multilayer perceptron (MLP) to learn the relationship between the lossy compressions U and *Y*.

Over the course of a training run, the coefficient of the IB strength *β* was swept to obtain a spectrum of compression schemes and predictive models. The distributed information plane (Fig. 1*C*) (16) displays the predictive power as a function of the total information about the inputs $\sum I(U_{i};X_{i})$. The predictive performance ranged from zero predictive information without any information about the inputs (Fig. 1*C*, *Lower Left*) to all entropy H(Y) accounted for by utilizing all ten bits of input information (Fig. 1*C*, *Upper Right*). For every point on the spectrum, there was an allocation of information over the inputs; the distributed IB objective identified the information across all inputs that was most predictive. The most predictive information about *Y* was found to reside in $X_{3}$—the input that routes through the fewest gates to *Y*—and then in the pair $X_{3},X_{10}$, and so on. Inputs that perform identical roles in the circuit were compressed nearly identically, as seen in the pairs $X_{1}$ and $X_{2}$, $X_{4}$ and $X_{5}$, and $X_{8}$ and $X_{9}$.

Machine learning facilitated the traversal of the space of lossy compression schemes of $X_{i}$, decomposing the information held within the circuit inputs about the output. Contained within the space of compression schemes are $2^{10}$ schemes that transmit complete information about discrete subsets of the inputs. To be concrete, there are ten subsets of a single input corresponding to compression schemes that transmit one bit of information about the input gates, 45 pairs of inputs that transmit two bits, and so on, with each subset sharing mutual information with *Y* based on the role of the specific inputs inside the circuit. Fig. 1*D* displays the information contained in every discrete subset of inputs (black points) along with the continuous trajectory found by optimization of the distributed IB (gray curve). The distributed IB, maximizing predictive information while minimizing information taken from the inputs, closely traced the upper boundary of discrete information allocations and identified a majority of the most informative subsets of inputs. To decompose the information in the circuit’s inputs required only a single sweep with the distributed IB, not an exhaustive search through all subsets of inputs. We note that the product of the distributed IB is not an ordering of single variable mutual information terms $I(X_{i};Y)$, which would be straightforward to calculate, but instead the ordering of information selected from all of X that is maximally informative about *Y*. Analysis of additional Boolean circuits with the distributed IB as well as comparisons to feature importance derived from logistic regression and Shapley values (15, 36, 37) may be found in *SI Appendix*.

### 2.2. Decomposing Structural Information in a Physical System.

Linking structure and dynamics in amorphous materials—complex systems consisting of particles crowded together in a disordered configuration—has been a longstanding challenge in physics (38–40). In particular, the deformation of amorphous materials generally proceeds in fits and starts, punctuated by sudden, localized rearrangement events reminiscent of earthquakes along a tectonic fault (24, 41, 42). Where, in the disordered configuration of the particles, is the information about whether a sudden rearrangement is about to occur? Searching for signatures of collective behavior in the multitude of microscopic degrees of freedom is an endeavor emblematic of the study of complex systems more generally and one well suited for machine learning and information theory. We accept that the functional relationship between the micro- and macroscale variation is potentially incomprehensible and are instead interested in the information at the microscale that is maximally predictive of behavior at the macroscale. While prior work has analyzed the information content of hand-crafted structural descriptors individually (43–45), the distributed IB searches through the space of information from many structural measurements in combination.

Two-dimensional simulated glasses, prepared by either rapid or gradual quenching and composed of small (type A) and large (type B) particles that interact with a Lennard–Jones potential, were subjected to global shear deformation (42). For each event, the location that precipitated the rearrangement was identified and paired with negative samples from elsewhere in the system to create a binary classification dataset.

We first characterized the microscale structure with a scheme of measurements that has been associated with plastic rearrangement in a variety of amorphous systems: the densities of radial bands around the center of a region (46, 47). By training a SVM to predict rearrangement based on the radial density measurements, a linear combination of the values is learned. In the literature, that combination is commonly referred to as softness, and has proven to be a useful local order parameter (48–51).

**Fig. 2.**
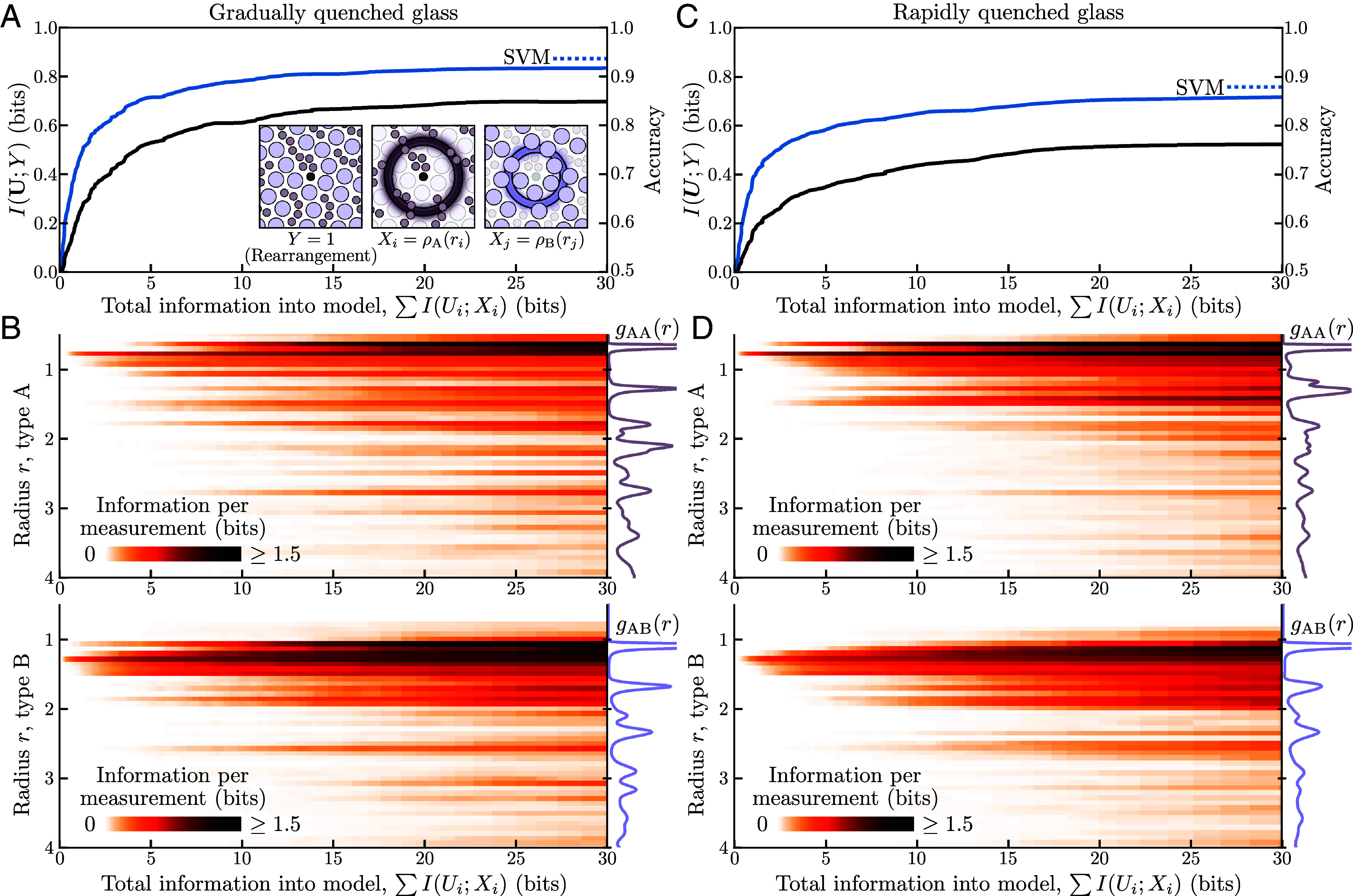
Decomposing structural information about imminent rearrangement in a sheared glass. (*A*) *Inset:* Given a local neighborhood in a sheared glass, densities of radial shells for the small (type A) and large (type B) particles were used to predict whether the neighborhood is the locus of an imminent rearrangement event. *Main:* For a gradually quenched glass, the information that is predictive of rearrangement (black) increased as the most predictive density information was identified and incorporated into the machine learning model. The accuracy (blue) was comparable to a support vector machine (SVM) (dashed line) after around twenty bits. (*B*) Sharing the horizontal axis with panel (*A*), the amount of information extracted about each of the radial density measurements of small (*Top*) and large (*Bottom*) particles reveals the radii with the most predictive information at each level of approximation. The system’s average density values for each particle type with type A at the center, also known as the radial distribution functions $g_{AA}(r)$ and $g_{AB}(r)$, are shown on the right. (*C* and *D*) The same as panels (*A* and *B*) but for glass that was prepared via a rapid quench rather than a gradual quench.

We approached the same prediction task from an information-theoretic perspective, seeking the specific bits of variation in the density measurements that are most predictive of collective rearrangement. Each radial density measurement underwent lossy compression by its own neural network before all compressions were concatenated and used as input to an MLP to predict rearrangement. By sweeping *β*, a single optimization recovered a sequence of distributed compression schemes, each allocating a limited amount of information across the 100 density measurements to be most predictive of imminent rearrangement (Fig. 2).

The trajectories in the distributed information plane (Fig. 2 *A* and *C*), for both gradually and rapidly quenched glasses, reflect the growth of predictive information and prediction accuracy given maximally predictive information about the radial densities. With only one bit of information from the density measurements, 71.8% predictive accuracy was achieved for the gradually quenched glass, and 69.5% was achieved for the rapidly quenched glass; with twenty bits, the accuracy jumped to 91.3% and 85.4%, respectively. Beyond twenty bits of density information, the predictive accuracy became comparable to that of the SVM, which can utilize all of the continuous-valued density measurements for prediction with a linear relationship.

For every point along the trajectory, information was identified from the density measurements that, together, formed the combination of bits that were most predictive of rearrangement. The allocation of information across the one hundred radial density measurements would be unintelligible if displayed in the distributed information plane as in Fig. 1*C*; instead, we employ heatmaps where each row corresponds to a different density measurement (Fig. 2 *B* and *D*). The majority of the information was selected from smaller radii (close to the center of the region), which can be expected given the localized nature of rearrangement events (38, 39). Less intuitive is the information decomposition as it relates to the radial distribution functions $g_{AA}(r)$ and $g_{AB}(r)$, the system-averaged radial densities of type A and B particles in regions with a type A particle at the center. For both glasses, the most predictive bits originated in the low-density radial bands nearest the center. As more information was incorporated into the prediction (moving left to right in the heatmaps), the additional bits came from radial bands that corresponded to particular features of $g_{AA}(r)$ and $g_{AB}(r)$. Outside of the first low-density region, the selected information often came from the high-density radii of type A particles and the low-density radii of type B particles; this trend held true for both glasses. While the information decomposition highlighted similar features in both glasses, the more pronounced structure of selected information out to larger radii for the gradually quenched glass is indicative of its higher structural regularity, which is also seen in the pronounced features of its radial distribution functions $g_{AA}(r)$ and $g_{AB}(r)$. For comparison, the weights of the trained SVM and estimated Shapley values may be found in the *SI Appendix*.

**Fig. 3.**
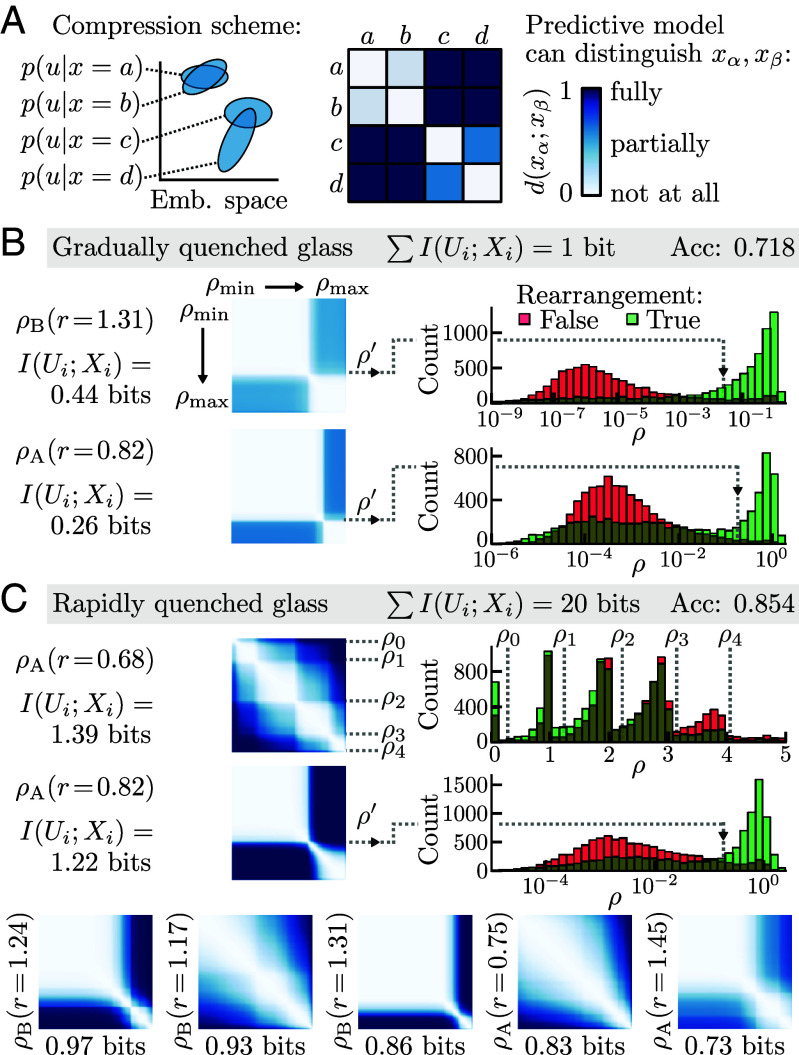
Selected bits of information as distinctions among raw measurement values. (*A*) Lossy compression is achieved by mapping the raw values of *X* to probability distributions in latent space. The statistical similarity of the conditional distributions, visualized as a distance matrix for all pairs of feature values, determines how distinguishable the raw feature values are to the predictive model. (*B*) The single most predictive bit of information about rearrangement in the gradually quenched glass came predominantly from two density measurements. The distinguishability matrices indicate that the compression scheme applied a simple threshold to these measurements: Density values less than a cutoff value $\rho^{\prime }$ were indistinguishable from each other, as were values above the cutoff. The histograms of density values conditioned on rearrangement (*Right*) show that the learned cutoff value separates the probability masses. (*C*) The twenty most predictive bits of radial density information in the rapidly quenched glass were selected from many radial bands. The two that contribute more than a bit of information each correspond to the density of type A particles near the center; one compression scheme effectively counted the number of particles in the high-density shell. The distinguishability matrices of the next five most informative radial bands are shown below.

The amount of information extracted from each density measurement was predominantly a single bit or less. In contrast to the Boolean variables in Fig. 1*C*, where $I(U_{i};X_{i})$ completely characterized the compression scheme, a continuous-valued density can be compressed to a bit in any number of ways. What was the specific variation extracted from each density measurement? Through inspection of the learned compression schemes, the extracted information can be further decomposed by the degree of distinctions between measurement values that were conveyed to the predictive model (Fig. 3*A*) (16). Serving to visualize a compression scheme, a distinguishability matrix is a distance matrix between values $x_{\alpha }$ and $x_{\beta }$ where a value of 0 indicates the states are indistinguishable, a value of 1 means that they are fully distinguishable, and an intermediate value means they are partially distinguishable. We found that the single most important bit of information for the gradually quenched glass (Fig. 3*B*) was a composition of partial bits from multiple density measurements, mostly from the first low-density shell of each type of particle. For both measurements, the compression scheme acted as a threshold on the range of possible density values: Values less than a cutoff $\rho^{\prime }$ were indistinguishable from each other for the purposes of prediction and were partially distinguishable from density values above the cutoff. By examining the distribution of density values in these radial shells, we see that the cutoff values leverage the separability of the density distributions when conditioned on rearrangement.

With more information utilized for prediction, some of the compression schemes differed from simple thresholds (shown for the rapidly quenched glass in Fig. 3*C*). For the predictive model operating with a total of twenty bits of density information, two density measurements contributed more than a bit each. The learned compression of the first high-density shell of type A particles essentially counted the number of particles in the shell, with distinguishability between densities as if there were several thresholds over the range of the values that act to roughly discretize the density measurement.

**Fig. 4.**
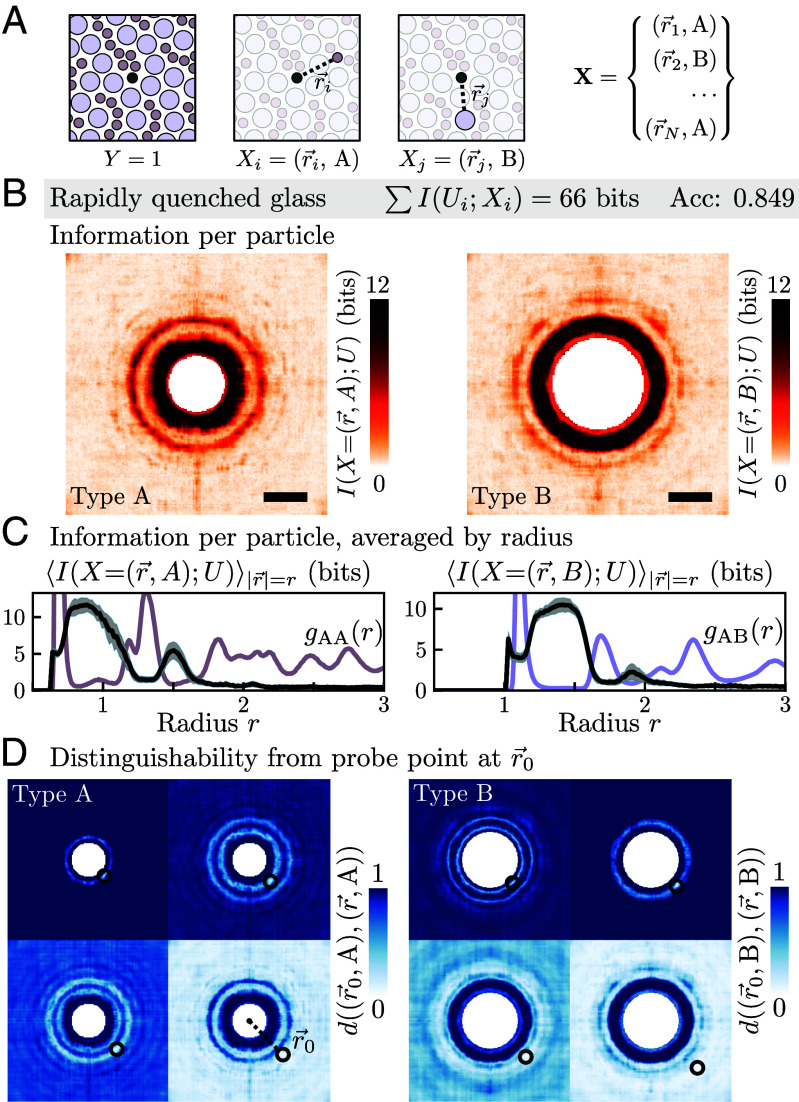
Information decomposed in terms of per-particle measurement basis. (*A*) Instead of the density of radial shells, each particle’s position and type in a local neighborhood were used as input measurements to relate to rearrangement. (*B*) The per-particle information transmitted as a function of particle position, for the small type A (*Left*) and large type B (*Right*) particles, for the predictive model utilizing 66 bits of information about the rapidly quenched glass. The scale bar is a distance of one in simulation units, equal to the length scale of interaction between types A and B particles. (*C*) Averaged radially, the information (black) resides in particles that are situated in the first troughs of the radial distribution function, g(r) (colored curves). (*D*) For a particle at position ${\vec{r}}_{0}$, the distinguishability of particles of the same type at all other locations has a radial structure and indicates that negligible azimuthal information was transmitted.

Decomposing the information in a composite X depends upon its basis of measurements (52). In the study of complex systems, there can be multiple “natural” schemes of measuring a system state. Measurements of the densities of radial bands lead to an essentially linear relationship between structure and rearrangement (48); what if we had not inherited such a fortuitous measurement scheme? Another natural basis of measurements is the position of all of the particles (Fig. 4*A*). To be clear, instead of representing a system’s local configuration in terms of one hundred measurements of radial density, we could have directly used the *N* measurements of the position and type of the *N* particles in a local region. In the preceding analysis, each radial density was considered a distinct information source and a unique compression channel was learned for each. In the per-particle measurement basis, there is no straightforward means to divide the measurements into separate sources because positions exist along a continuum; accordingly, a single compression channel was used for each type, compressing in parallel every particle of type A and then similarly for type B. To subsequently predict rearrangement based on the set of compressed per-particle measurements, which lack a canonical ordering, we used a permutation-invariant transformer architecture (53).

The per-particle measurement scheme imposed no positional structure on the selection of configurational information. Nevertheless, we found that the information cost per particle as a function of the position in the neighborhood had a radial structure (Fig. 4*B*). The information per particle was highest in the low-density radial bands near the center of the region (Fig. 4*C*), and inspection of the compression scheme indicated that negligible azimuthal information was transmitted (Fig. 4*D*). The information decomposition allowed for similar insights to be derived as in the radial density measurement scheme, even though the nature of the predictive model in the two cases was substantially different. Additionally, because the distributed IB operates entirely on the input side of an arbitrary predictive model, the information analysis was agnostic to whether the model was a simple fully connected network or a more complicated transformer architecture.

## 3. Discussion

A universal challenge faced when studying complex systems, fundamental to what makes a system complex, is the abundance of entropy from the perspective of the microscale that obscures relevant information about macroscale behavior. The generality of mutual information as a measure of statistical relatedness, and the capacity of deep learning to handle high-dimensional data, allow the distributed IB to be as readily utilized to identify structural defects relevant to a given material property as it is to reveal gene variation relevant to a given affliction. Tens, hundreds, and potentially thousands of measurements of a complex system are handled simultaneously, rendering practical analyses that would have previously been infeasible through exhaustive search or severely limited by constraints on functional relationships between variables.

Information theory has long held appeal for the analysis of complex systems owing to the generality of mutual information (1, 29). However, the estimation of mutual information from data is fraught with difficulties (34, 35, 54), and the rapid growth in ways information can be distributed across a number of variables (55) have hindered information-theoretic analyses of data from complex systems. By distributing IBs across multiple partial measurements of a complex system, entropy is partitioned to a degree that makes precise estimation of mutual information possible while simultaneously revealing the most important combinations of bits for insight about the system. Machine learning navigates the space of lossy compression schemes for each variable and allows the identification of meaningful variation without consideration of the black box functional relationship found by the predictive model.

Instead of compressing partial measurements in parallel, the IB (19) extracts the relevant information from one random variable in its entirety about another and is foundational to many works in representation learning (56, 57). In the physical sciences, the IB has been used to extract relevant degrees of freedom with a theoretical equivalence to coarse-graining in the renormalization group (58, 59), and to identify useful reaction coordinates in biomolecular reactions (60). However, the IB generally has limited interpretability because the singular bottleneck occurs after processing the complete input, allowing the compression scheme to involve arbitrarily complex relationships between components of the input without penalty. Additionally, when the relationship between X and *Y* is deterministic (or nearly so), the entire spectrum of extracted information is trivially a copy of *Y* with added noise (52, 61, 62). The distribution of IBs across observables is critical to an interpretable information decomposition and to recovering an illuminating spectrum of extracted information.

A growing body of literature focuses on a fundamentally different route to decompose the information contained in multiple random variables $\left\{X_{i}\right\}$ about a relevant random variable *Y*; that alternative route is partial information decomposition (PID) (55, 63). Although there is no consensus on how to achieve PID in practice, its goal is to account for the mutual information between $\left\{X_{i}\right\}$ and *Y* in terms of subsets of $\left\{X_{i}\right\}$, by analogy to set theory (64). PID allocates information to the input variables in their entirety, whereas the distributed IB selects partial entropy from the input variables in the form of lossy compression schemes, with one scheme per variable. While PID has been proposed as an information-theoretic route to study complex systems (65) and quantify complexity (66), the superexponential growth of PID terms renders the methodology somewhat impractical unless “coarse-grained” metrics are used (10, 67). There are $5\times 10^{22}$ PID terms for a Boolean circuit with 8 inputs (55) and the number of terms for the 10-input circuit from Fig. 1 is not known (63). By contrast, the distributed IB offers a pragmatic route to the decomposition of information in a complex system: It is amenable to machine learning and data and can readily process one hundred (continuous) input variables as in the amorphous plasticity experiments. Although the distributed IB navigates a different space of information terms than those induced by PID, it may prove fruitful to employ the former as a means to focus on the most significant terms of the latter.

## Supplementary Material

Appendix 01 (PDF)

Dataset S01 (GZ)

## Data Availability

The glass dataset is attached to this paper and has also been deposited to Figshare (68) (https://doi.org/10.6084/m9.figshare.24585150.v1). The code base, including code to generate truth tables from Boolean circuits, has been posted to GitHub and may be found through the following link: https://distributed-information-bottleneck.github.io.
