# Supplementary material for: Information decomposition in complex systems via machine learning
Source: Proc Natl Acad Sci U S A. 2024 Mar 18;121(13):e2312988121. doi: 10.1073/pnas.2312988121 (PMC10990158; doi:10.1073/pnas.2312988121)
Supplement: Supplementary file 1 — Appendix 01 (PDF) [file pnas.2312988121.sapp.pdf]

Supporting Information for

Information decomposition in complex systems via machine learning

Kieran A. Murphy and Dani S. Bassett

Dani S. Bassett.
E-mail: dsb@seas.upenn.edu

This PDF file includes:

Supporting text
Figs. S1 to S4
Legend for Dataset S1
SI References

Other supporting materials for this manuscript include the following:

Dataset S1

Supporting Information Text

Code availability. The full code base has been released on Github and may be found through the following link: distributed-information-bottleneck.github.io. Every analysis included in this work can be repeated from scratch with the corresponding Google Colab iPython notebook in this directory.

Boolean circuitry: Comparative analyses and extended examples

To further develop intuition about the manner of information decomposition achieved by the distributed IB, we analyze several additional Boolean circuits in this section. As a reminder, the distributed IB ingests a dataset of input-output observations and yields a decomposition of the information contained in the inputs about the output. The decomposition offers a degree of interpretability about the relationships between the inputs so far as they determine the output. The ground truth circuitry in these examples is used to create the data but then does not factor into the information decomposition; it is displayed only as a point of reference.

First, we randomly generated Boolean circuits with three to six input gates (Fig. S1). To serve as reference analyses, we analyzed data from the circuits with a linear and a nonlinear method that each provide a sense of the importance of the inputs X_i in determining the output Y. We performed logistic regression and computed the Shapley values in relation to the mutual information between $\{X_i\}$ and Y (1–3) (Fig. S1, left column). We then optimized the distributed IB following the training protocol of Fig. 1 of the main text (Fig. S1, middle column). We again compared the distributed IB decomposition to the exhaustive set of mutual information terms, where there is one for each possible subset of inputs (Fig. S1, right column). By contrast to Fig. 1 of the main text, the circuits are small enough to allow visualization of the specific input combination (shown as pie charts) represented by each point in the plot.

In **logistic regression**, a linear combination of the inputs is fit in log-probability space to be maximally predictive of the label Y, and this linearity grants interpretability to the model weights. However, the linearity also severely restricts the relationships that can be modeled. In the six circuits in Fig. S1, the nonlinearity of `XOR` gates diminished predictive power of the logistic models: the amount of information captured by the model, $I(f(X);Y)$, was less than 0.2 bits for four of the circuits. In the best case (the circuit in panel (e), with only one out of four gates being `XOR`), the model captured 0.75 bits of information about Y and had an accuracy of 94%. Despite the inability to represent the nonlinearity of `XOR`, there was some degree of success in shedding light on the inner workings of the circuit: the model coefficients revealed one or a few of the most informative inputs in circuits (b), (c), (d), and (e).

Shapley values (3) have become a popular approach to explaining machine learning models because they convey a sense of importance of inputs (or features) with regard to an output while being agnostic to the model used. Originating in game theory as a way to assign credit to a set of players in a game, the most common formulation in explainable ML (1) grants *local* interpretability (3) to a trained model; i.e., it provides case-by-case explanations of model outputs for individual examples from the data. By contrast, the distributed IB provides *global* interpretability (4), meaning the insights pertain to the entire data distribution (and the entire system) at once. Instead of assigning credit for a single model prediction, Shapley values can be computed with regard to the information gain of a model in order to provide global interpretability (2). We use the truth table for each circuit as a perfect model, though we could also have trained a neural network and estimated Shapley values following Covert *et al.* (2). The Shapley value for each input is a weighted sum of all possible mutual information terms (the same terms displayed in the right column of Fig. S1), serving to summarize the contribution of information for each input in the context of every combination of other inputs. A desirable property of Shapley values is that they are additive: for the deterministic relationships represented by these circuits, the Shapley values for all inputs sum to the entropy $H(Y)$ of the output.

The Shapley values relative to information gain conveyed more about the underlying circuitry than the logistic model coefficients, which is sensible given that the "model" used for the Shapley values is the full truth table. We found that the values conveyed the same high-level feature importance as the distributed IB, successfully accounting for higher-order interaction effects between the gates because the information in all subsets of inputs is included in the calculation.

In contrast to logistic regression and Shapley values, which produce a single value of importance per input, the distributed IB produces an entire spectrum of importance values in the form of information allocations across the inputs. The spectrum represents a Pareto frontier, where every point in the spectrum contains the most information about the output Y for the least total information about the inputs X_i. The allocations order the information contained in the inputs from most to least relevant about the output Y. By reading the distributed information plane from left to right (middle column of Fig. S1), we infer the importance of the inputs by the order in which they appear in the information allocations. The rank order of the importance of the inputs matches that of the Shapley values in every circuit. What else can be learned from the distributed IB by way of the spectrum of information allocations?

Consider the three-input circuits of Fig. S1 (a) and (b). Given only the Shapley values for the three inputs in panel (a) we infer that the inputs are equally responsible for determining the output, but little else is learned. With the information decomposition from the distributed IB, we learn far more about the circuit. The identical trajectories of the inputs in the distributed information plane suggests the inputs perform equivalent roles. The slow growth of the predictive information $I(\boldsymbol{U};Y)$ suggests a highly entangled interaction between the inputs whereby substantial information about all three is required before any information is gleaned about Y. By comparison, the growth of predictive information for the circuit in panel (b), which increases immediately with information about X_3, tells us that information about Y is available with information about only the third input.

Consider again the three curves displaying the information allocation to the inputs in the distributed information plane of Fig. S1b. One (corresponding to X_3) is concave while the other two (X_1 and X_2) are convex and mirror each other; the curves tell about the growth of information of the inputs in combination. Similar behavior can be seen in Fig. S1c with X_4, X_1 and X_3; inspection of the circuits shows there is an `AND-XOR` subcircuit common to the circuits in both panels (b) and (c). By chance (because the circuits were randomly sampled) the replication can be taken one step further. The entire information allocation of the circuit in Fig. S1c is found in the circuit in Fig. S1e, and again the circuits can be observed to share a subcircuit. The information allocations and the growth of predictive information tell much more about the interactions between inputs than can be conveyed in a single list of importance values.

In the right column of Fig. S1, we display the information about Y contained in all possible subsets of inputs, allowing a check on the quality of information allocations recovered by the distributed IB. As in the main text, we again found that the distributed IB was able to identify the most informative subsets without requiring exhaustive search. Notably, for a circuit of only `XOR` gates (Fig. S1a), the distributed IB outperformed the discrete allocations by leveraging partial information allocations. With only discrete information allocations across the inputs, there is a discontinuous transition from zero information about Y with knowledge of any two input gates, to complete information about Y when knowing all three. In stark contrast, by exploring the larger space of soft compression schemes (in which partial information can be transmitted), machine learning found a smooth interpolation between zero and complete information about Y.

Comparison of the space of compression schemes relevant to the distributed and standard IB. In Fig. S2 we analyzed a single `XOR` logic gate with both the distributed IB and the standard IB. For the distributed IB (Fig. S2a), each of the inputs X_1 and X_2 was compressed to its own variable, U_1 and U_2, defined by the conditional distributions $p(u_1|x_1)$ and $p(u_2|x_2)$. We sampled from the space of distributed compression schemes by randomly sampling conditional distributions over a two-symbol alphabet for each U_i. 2500 distributed compression schemes are plotted as gray dots in the distributed information plane in Fig. S2a, along with the trajectory navigated by the distributed IB. The convex shape of the distributed IB trajectory closely traced the boundary of distributed compression schemes.

By contrast to the distributed compression schemes, the standard IB compresses the entire input $\boldsymbol{X}$ to a single variable U and optimizes the IB Lagrangian (5)

$$\mathcal{L}_{\mathrm{IB}} = \beta I(U;\boldsymbol{X}) - I(U;Y). \quad [1]$$

We sampled from the space of compression schemes by randomly sampling conditional distributions over a four-symbol alphabet for U (Fig. S2b). In total, 15000 compression schemes were created by sampling the sixteen values of the relevant conditional distributions from a uniform distribution and raising to the sixth power before normalizing. We found that sampling probability values uniformly (i.e., without exponentiation) rarely sampled compression schemes with $I(U;\boldsymbol{X}) \gtrsim 1$ bit. With the same variational bounds we used for the distributed IB (6), we optimized the standard IB and found that it successfully traced the boundary of relevant compression schemes.

The space of distributed compression schemes is more restrictive than the space of compression schemes relevant to the standard IB. Because the components of $\boldsymbol{X}$ are independent for this example, the two horizontal axes of Fig. S2a,b are equivalent, allowing the point scatter of compression schemes to be directly compared. While the distributed schemes in Fig. S2a are included in the space of schemes in Fig. S2b, only the latter includes schemes that utilize knowledge of the full state of $\boldsymbol{X}$. As an example, one scheme that optimizes the standard IB maps $\boldsymbol{X} \in \{01, 10\}$ and $\boldsymbol{X} \in \{00, 11\}$ to two separate clusters, effectively extracting Y without revealing anything about the structure of $\boldsymbol{X}$—a shortcoming of the standard IB when analyzing deterministic relationships (7).

Glassy rearrangement: Comparative analyses of radial density measurements

For the simple Boolean circuits of Fig. S1, all three analyses—logistic regression, Shapley values, and the distributed IB—conveyed a sense of importance of X_i in determining Y. The unique capabilities of the distributed IB become more apparent under more challenging scenarios, such as relating rearrangement in a simulated glass to local radial density measurements. In Fig. S3, we reproduce from Fig. 2 of the main text the distributed IB information decomposition and compare it to the weights of a support vector machine (SVM) as well as the Shapley values estimated for an MLP[*] (following a method named SAGE (2)[†]). There is general agreement about the most important radial bands as told by the order of information allocation by the distributed IB, and the magnitude of weights derived from the (linear) SVM and the (nonlinear) Shapley values. The distributed IB reveals far more, however: all of the most informative subsets of radial bands and the specific bits of information from each radial density that are relevant to rearrangement (Fig. 3 of the main text). To find the most informative subsets of radial bands, the authors of (8) trained millions of SVMs, each on different subsets of the radial densities—quickly making exhaustive evaluation impractical beyond a handful of measurements.

Regarding the specific bits of relevant information (visualized by the distinguishability matrices of Fig. 3 in the main text), we are aware of no method other than the distributed IB that can do so while modeling nonlinear relationships with the full expressivity of deep learning. Finally, we note that the per-particle measurement basis (Fig. 4 of the main text) similarly has no apparent analogue in terms of Shapley values. With the distributed IB we are able to inspect the compression channel and study the information allocated to hypothetical particles, even without a well-defined set of features as would be required for analysis by methods that assess feature importance.

[*] Two layers of 256 `Leaky ReLU` units, trained for 10 epochs.

[†] https://github.com/iancovert/sage

Mutual information bounds

Bounding mutual information given high-dimensional data is notoriously difficult (9, 10). Fortunately, there are factors in our favor to facilitate optimization with machine learning and, during evaluation, the recovery of tight bounds on the information transmitted by the compression channels U_i.

During training, to optimize the distributed information bottleneck objective requires a lower bound on $I(\boldsymbol{U};Y)$ and an upper bound on $I(U_i;X_i)$. For $I(\boldsymbol{U};Y)=H(Y)-H(Y|\boldsymbol{U})$, we use the cross entropy loss of the predictions as a lower bound on $H(Y|\boldsymbol{U})$ and ignore $H(Y)$ because it is constant (6). Regarding $I(U_i;X_i)$, the (distributed) variational information bottleneck objective (6, 11) upper bounds $I(U_i;X_i)$ with the expectation of the Kullback-Leibler (KL) divergence between the encoded distributions $p(u_i|x_i)$ and an arbitrary prior distribution $r(u_i)$ in latent space,

$$I(U_i;X_i) \le \mathbb{E}_{x_i\sim p(x_i)}[D_{\mathrm{KL}}(p(u_i|x_i)||r(u_i))]. \quad [2]$$

Normal distributions are used for both the encoded distribution, $p(u_i|x_i)=\mathcal{N}(\boldsymbol{\mu}=f_\mu(x_i),\boldsymbol{\sigma}=f_\sigma(x_i))$, and the prior, $r(u_i)=\mathcal{N}(\boldsymbol{0},\boldsymbol{1})$ so that the KL divergence has a simple analytic form.

For evaluation over the course of a training run, the KL divergence is computed for each channel U_i in the process of computing the loss, and could be used for a qualitative sense of information allocation to features (4). However, the KL divergence is a rather poor estimate of the mutual information, and we seek to know the amount of transmitted information as precisely as possible. Because the encoded distributions $p(u_i|x_i)$ have a known form, we can use the noise contrastive estimation (InfoNCE) lower bound and "leave one out" upper bound from Ref. (12) with a large number of samples to obtain tight bounds on the amount of mutual information in the learned compression schemes (for evaluation).

The lower and upper bounds on $I(U_i;X_i)$ are based on likelihood ratios at points sampled from the dataset $x_i\sim p(x_i)$ and from the corresponding conditional distributions, $u_i\sim p(u_i|x_i)$. To be specific, the mutual information for each channel $U=f(X)$ (dropping channel indices for simplicity) is lower bounded by

$$I(U;X) \ge \mathbb{E}\left[\frac{1}{K}\sum_i^K \log\frac{p(u_i|x_i)}{\frac{1}{K}\sum_j^K p(u_i|x_j)}\right] \quad [3]$$

and upper bounded by

$$I(U;X) \le \mathbb{E}\left[\frac{1}{K}\sum_i^K \log\frac{p(u_i|x_i)}{\frac{1}{K-1}\sum_{j\neq i}^K p(u_i|x_j)}\right]. \quad [4]$$

The expectation values in both equations are taken over samples $\{u_i,x_i\}_{i=1}^K$ of size K extracted repeatedly from the joint distribution $p(u,x)=p(x)p(u|x)$. We estimated with as large an evaluation batch size K as feasible given memory and time considerations, and then averaged over multiple batches to reduce the variance of the bound. Evaluation with a batch size of 1024, averaged over 8 draws, yielded bounds on the mutual information that was on the order of 0.01 bits for the Boolean circuit and glass data. The size of the validation dataset for the glass and the size of the truth table of the Boolean circuit were both on the order of one thousand points. Hence, the benefit of averaging comes from repeated sampling of the latent representations.

We show in Fig. S4 the performance of the mutual information bounds for compression schemes that encode up to several bits of information. X is a discrete random variable that is uniformly distributed over its support and has one to six bits of entropy; for each X a fixed dataset of size 1024 was sampled for mutual information estimation according to the following method of compression. Each outcome x was encoded to a normal distribution with unit variance in 32-dimensional space, $p(u|x)=\mathcal{N}(\boldsymbol{\mu},\boldsymbol{1})$. The encoded distributions were placed along orthogonal axes a distance d from the origin; in the limits of $d=0$ and $d\gg 1$ the information transmitted by the compression scheme is 0 and $H(X)$, respectively.

A Monte Carlo estimate of the mutual information sampled 2×10^5 points from $p(u,x)$ to compute $\mathbb{E}_{p(u,x)}[\log p(u|x)/p(u)]$. The "leave one out" upper and InfoNCE lower bounds were computed with different evaluation batch sizes K, and averaged over 4096 sampled batches. The standard deviation of the bounds is displayed as the shaded region around each trace, and is left out of the plots for the residual (the difference between the bound and the Monte Carlo estimate) for all but the evaluation batch size of 1024.

When the information contained in the compression is less than about two bits—as was the case for the majority of the experiments of the main text—the bounds are tight in expectation for even the smallest evaluation batch size. The variance is reducible by averaging over multiple batches. As the transmitted information grows, the benefit of increasing the evaluation batch size grows more pronounced, though bounds with a range of less than 0.1 bits can still be achieved for up to six bits of transmitted information.

Information transmitted per particle. For the per-particle measurement scheme on the glass data, a single compression channel U was used for all particles. The information conveyed by the channel $I(U;X)$ may be estimated as above, with X being the particle position and type. Note that we are particularly interested in the information cost for specific particle positions and for each particle type. The outer summation of the bounds (Eqn. 3 and 4) serves to average over the measurement outcomes x_i in a random sample; we use the summand corresponding to $\{x_i,u_i\}$ as the information contribution for the specific outcome x_i. To generate the information heatmaps of Fig. 4b in the main text, we randomly sampled 512 neighborhoods from the dataset, corresponding to an evaluation batch size $K=512$ neighborhoods $\times$ 50 particles / neighborhood $=25,600$ particles

(data points), and averaged over 100 such batches. A probe particle with specified particle type and position (one for each point in the grid) was inserted into the batch, and then the corresponding summand for the lower and upper information bounds served to quantify the information transmitted per particle. To be specific,

$$I(X=x;U) \geq \mathbb{E}\left[\log \frac{p(u|x)}{\frac{1}{K}\sum_j^K p(u|x_j)}\right], \qquad [5]$$

with the expectation taken over $u \sim p(u|x)$ and samples $\{x_i\}_{i=1}^K \sim \prod_i^K p(x)$. The upper bound differed only by inclusion of the distribution $p(u|x)$ corresponding to the probe point in the denominator's sum.

Implementation specifics

All experiments were implemented in TensorFlow and run on a single computer with a 12 GB GeForce RTX 3060 GPU. Computing mutual information bounds repeatedly throughout an optimization run contributed the most to running time. Including the information estimation, the Boolean circuit optimization took about half an hour, and the glass experiments took several hours.

Boolean circuit. Each input may take only one of two values (0 or 1), allowing the encoders to be extremely simple. Trainable scalars $(\vec{\mu}_i, \log \vec{\sigma}_i^2)$ were used to encode $p(u_i|x_i) = \mathcal{N}((2x_i-1)\times\vec{\mu}_i, \vec{\sigma}_i^2)$. The decoder was a multilayer perceptron (MLP) consisting of three fully connected layers with 256 `Leaky ReLU` units ($\alpha = 0.3$) each. We increased the value of β logarithmically from 5×10^{-4} to 5 in 5×10^4 steps, with a batch size of 512 input-output pairs sampled randomly from the entire 1024-element truth table. We found the information decomposition to be insensitive to annealing duration as long as it was around 5×10^4 steps or longer. The Adam optimizer was used with a learning rate of 10^{-3}.

Amorphous plasticity. The simulated glass data comes from Ref. (13): 10,000 particles in a two-dimensional cell with Lees-Edwards boundary conditions interact via a Lennard-Jones potential, slightly modified to be twice differentiable (14). Simple shear was applied with energy minimization after each step of applied strain. The critical mode was identified as the eigenvector—existing in the $2N$-dimensional configuration space of all the particles' positions—of the Hessian whose eigenvalue crossed zero at the onset of global shear stress decrease. The particle that was identified as the locus of the rearrangement event had the largest contribution to the critical mode (13).

We used data from the gradual quench ("GQ") and rapid quench (high temperature liquid, "HTL") protocols. Following Ref. (8), we considered only neighborhoods with type A particles (the smaller particles) at the center. We used all of the events in the dataset: 7,255 for the gradually quenched and 10,178 for the rapidly quenched glasses. For each rearrangement event with a type A particle as the locus, we selected at random another region from the same system state with a type A particle at the center to serve as a negative example. 90% of all rearrangement events with type A particles as the locus were used for the training set and the remaining 10% were used as the validation set; the regions and specific training and validation splits used in this work can be found on the project webpage.

Radial density measurement scheme. For the radial density measurements (Figs. 2,3 of the main text), the local neighborhood of each sample was processed using 50 radial density structure functions for each particle type, evenly spaced over the interval $r = [0.5, 4]$. Specifically, for particle i at the center and the set of neighboring particles $\mathcal{S}_A$ of type A,

$$G_A(i;r,\delta) = \sum_{j\in\mathcal{S}_A} \exp(-\frac{(R_{ij}-r)^2}{2\delta^2}), \qquad [6]$$

where R_{ij} is the distance between particles i and j. The same expression was used to compute G_B, the structure functions for the type B particles in the local neighborhood. The width parameter δ was equal to 50% of each radius interval.

After computing the 100 values summarizing each local neighborhood, the training and validation sets were normalized with the mean and standard deviation of each structure function across the training set. The best validation results from a logarithmic scan over twenty values from 10^{-3} to 10^1 for the C parameter were used for the value of the SVM accuracy in Fig. 2 of the main text.

For the distributed IB, each of the 100 scalar values for the structure functions were input to their own MLP consisting of 2 layers of 128 units with `tanh` activation. The embedding dimension of each U_i was 32. Then the 100 embeddings were concatenated for input to the predictive model, which was an MLP consisting of 3 layers of 256 units with `tanh` activation. The output was a single logit to classify whether the particle at the center is the locus of imminent rearrangement. We increased β in equally spaced logarithmic steps from 10^{-6} to 1 over 250 epochs (an epoch is one pass through the training data); these hyperparameters were selected based on peak validation performance. The batch size was 256. The Adam optimizer was used with a learning rate of 10^{-4}.

Per-particle measurement scheme. For the per-particle measurements, the nearest 50 particles to the center of each region were compressed by the same encoder, an MLP with two layers of 128 `Leaky ReLU` activation (α=0.1), to a 32-dimensional latent space. The only information available to the encoder was the particle's position and type, though the values were preprocessed before input to the encoder to help with optimization: for each particle position $\vec{r} = (x, y)$, we concatenated x^2, y^2, $r = |\vec{r}|$, $\log r$, $\log x^2$, $\log y^2$, and $\vec{r}/r$. All were positionally encoded (i.e., before being passed to the MLP, inputs were mapped to $x \leftarrow (x, \sin\omega_1 x, \sin\omega_2 x, ...))$ with frequencies $\omega_k = 2^k$, where $k \in \{1, 2, 3, 4, 5\}$ (4, 15).

After compression, the 50 representations (one for each particle) were input to a set transformer (16), a permutation-invariant architecture that is free to learn how to relate different particles via self-attention. We used 6 multi-head attention (MHA) blocks with 12 heads each, and a key dimension of 128. Following Ref. (16), each MHA block adds the output of multi-head attention to a skip connection of the block's input, and applies layer normalization to the sum. This intermediate output is passed through an MLP (a single layer with 128 `ReLU` units, in our case) and added to itself (another skip connection) before a second round of layer normalization. After the MHA blocks, the 50 particle representations were mean-pooled and passed through a final fully connected layer of 256 units with `Leaky ReLU` activation (α=0.1) before outputting a logit for prediction.

Training proceeded for 25,000 training steps, and the learning rate was ramped linearly from zero to 10^{-4} over the first 10% of training. Over the duration of training, β increased logarithmically from 3×10^{-8} to 3×10^{-3}. The batch size was 64.

Citation Diversity Statement

Science is a human endeavour and consequently vulnerable to many forms of bias; the responsible scientist identifies and mitigates such bias wherever possible. Meta-analyses of research in multiple fields have measured significant bias in how research works are cited, to the detriment of scholars in minority groups (17–21). We use this space to amplify studies, perspectives, and tools that we found influential during the execution of this research (22–25).

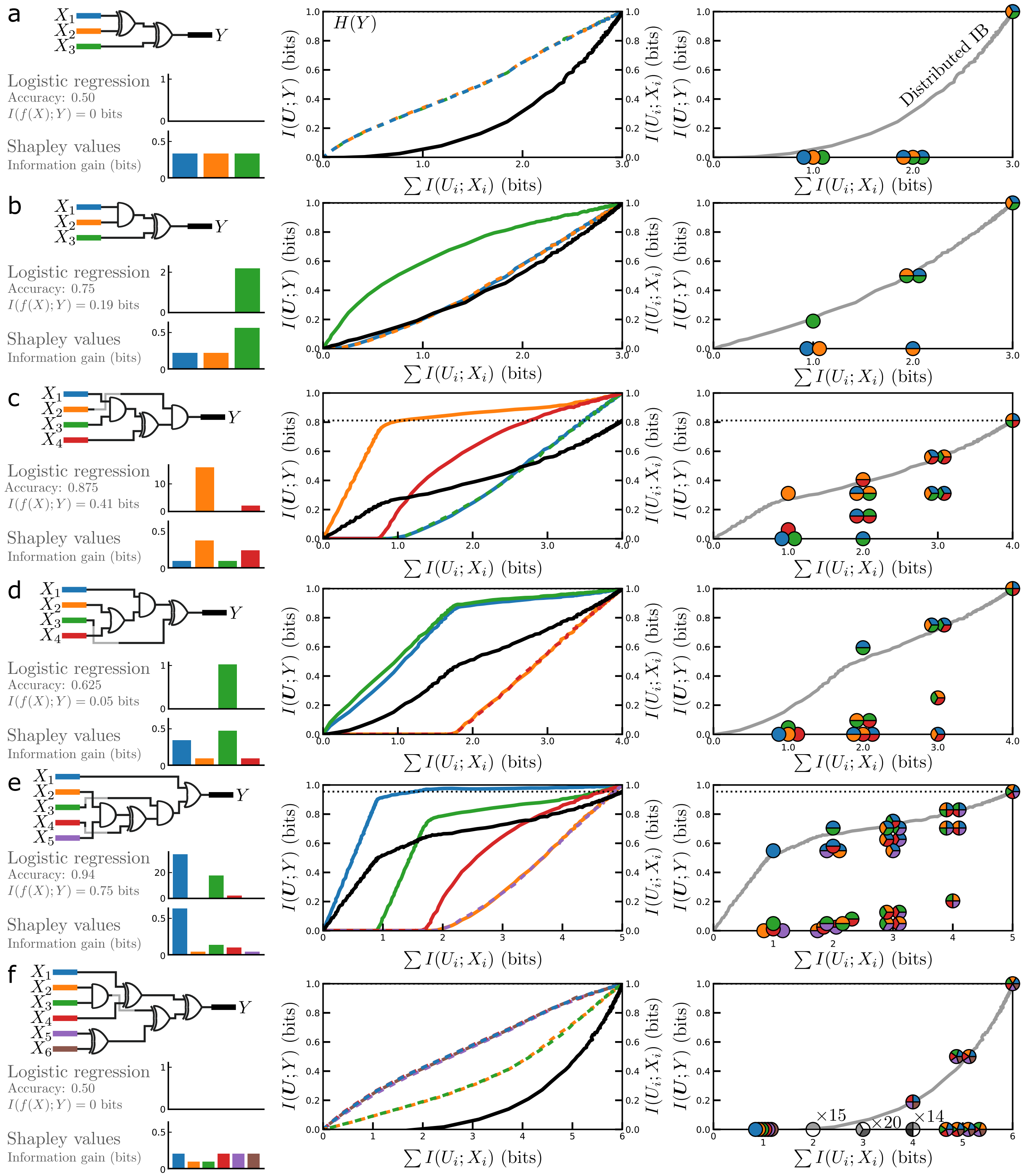

Fig. S1. Information decomposition of additional Boolean circuits. (a-f) Distributed IB analysis of randomly generated Boolean circuits, from three to six input gates. The circuit diagram (left) displays the circuit that was used to generate the joint distribution for training the distributed IB. Under each circuit we show two alternative routes to probe the importance of X_i with respect to Y: model weights of logistic regression and Shapley values regarding information gain about the output Y. The distributed information plane (middle) shows the total information $I(\boldsymbol{U};Y)$ (black) and the information allocation by input gate $I(U_i;X_i)$ (color) as a function of total information transmitted to the predictive model, $\sum I(U_i;X_i)$. Information allocation curves are dashed in cases where it aids visual clarity. On the right, we reproduce the distributed IB trajectory in gray and compare to the information contained by each of the possible discrete subsets. Each discrete subset is colored according to the input gates in the subset. We have shifted points horizontally when there was overlap. In all plots, the horizontal dashed line indicates $H(Y)$, the entropy of Y, though it occasionally coincides with the upper horizontal axis. In **f**, we suppress visualization of the subsets that all have zero information, and instead indicate the number of such subsets.

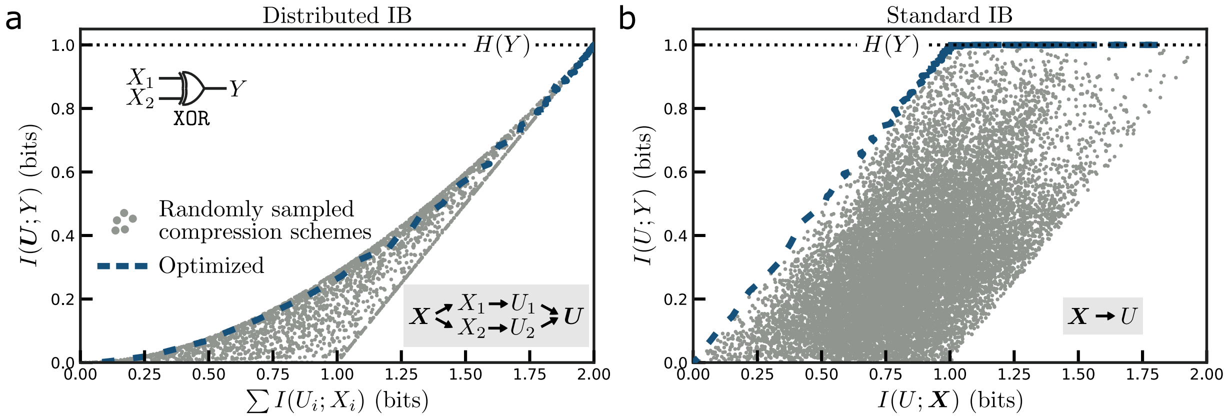

Fig. S2. The space of compression schemes navigated by the distributed and standard IB. (a) For a single XOR gate, the two inputs are compressed independently according to the distributed IB, in terms of relevance to the output Y. We randomly sample 2500 compression schemes (gray points) and additionally optimize the variational formulation of the distributed IB from the main text (blue, dashed). **(b)** With the same XOR gate as in **a**, the full input $\boldsymbol{X}$ is compressed to a single compression variable U. We randomly sample 15000 compression schemes (gray points) and additionally optimize the variational IB (blue, dashed). The space of distributed compression schemes in **a** is contained within the space of compression schemes of **b**. Because the components of $\boldsymbol{X}$ are independent in this example, the horizontal axes of **a** and **b** are equivalent.

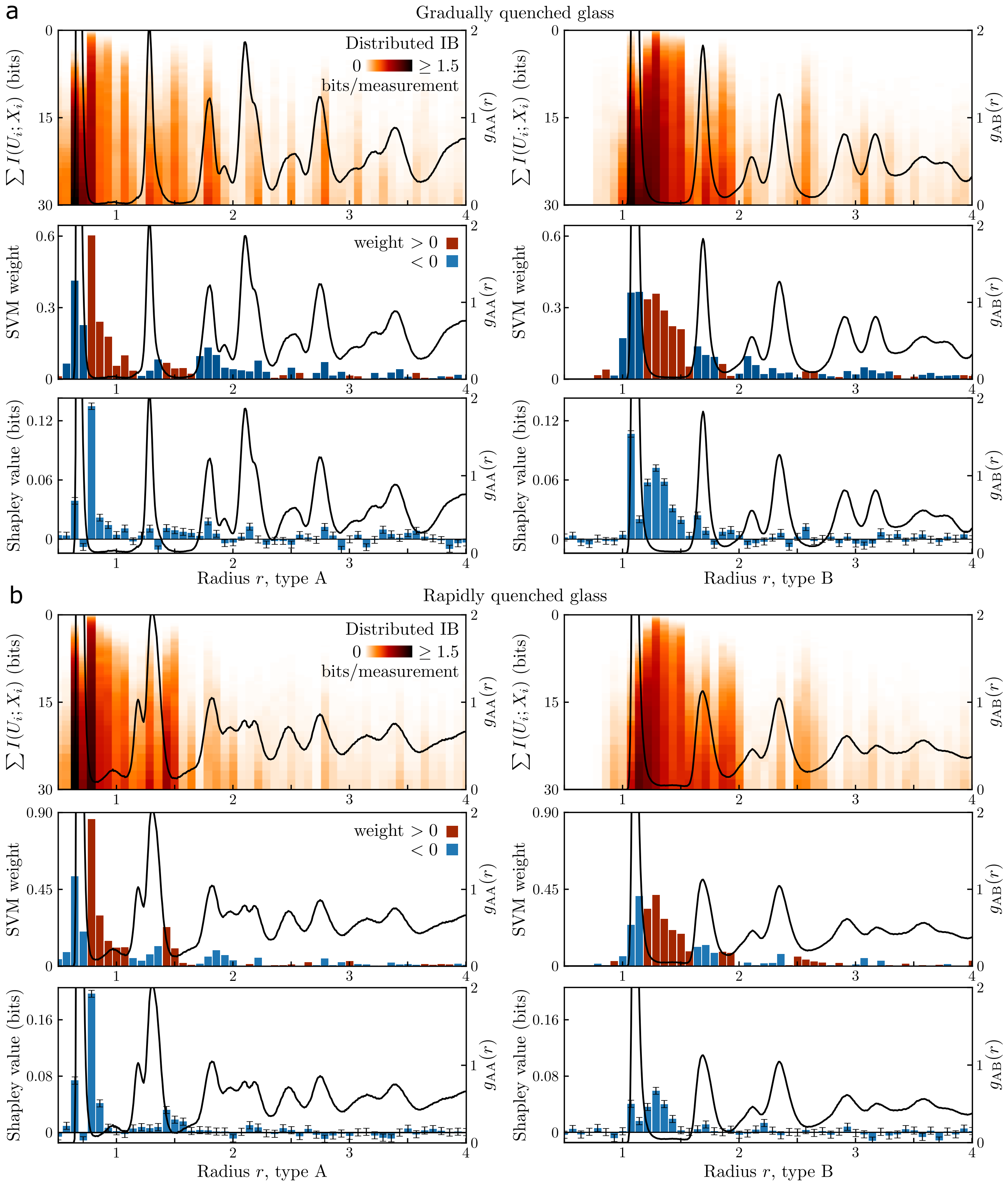

Fig. S3. Comparative analyses of radial density measurement scheme. (a) For the simulated glass prepared with a gradual quench, we reproduce the information allocation from the main text and compare to the weights of a support vector machine (SVM) and to the estimated Shapley values for the model's information gain. The absolute value of the SVM weights are shown, with red (blue) to indicate that the weight is positive (negative). The error bars on the Shapley values are estimated during sampling according to SAGE (2). The radial densities for type A particles are shown on the left and type B on the right, though we note that both sets of densities were used in combination as input to the models. The radial distribution functions $g_{AA}(r)$ and $g_{AB}(r)$, showing system-averaged radial densities, are the black curves and utilize the right vertical axes. **(b)** The same as **a**, for the rapidly quenched glass.

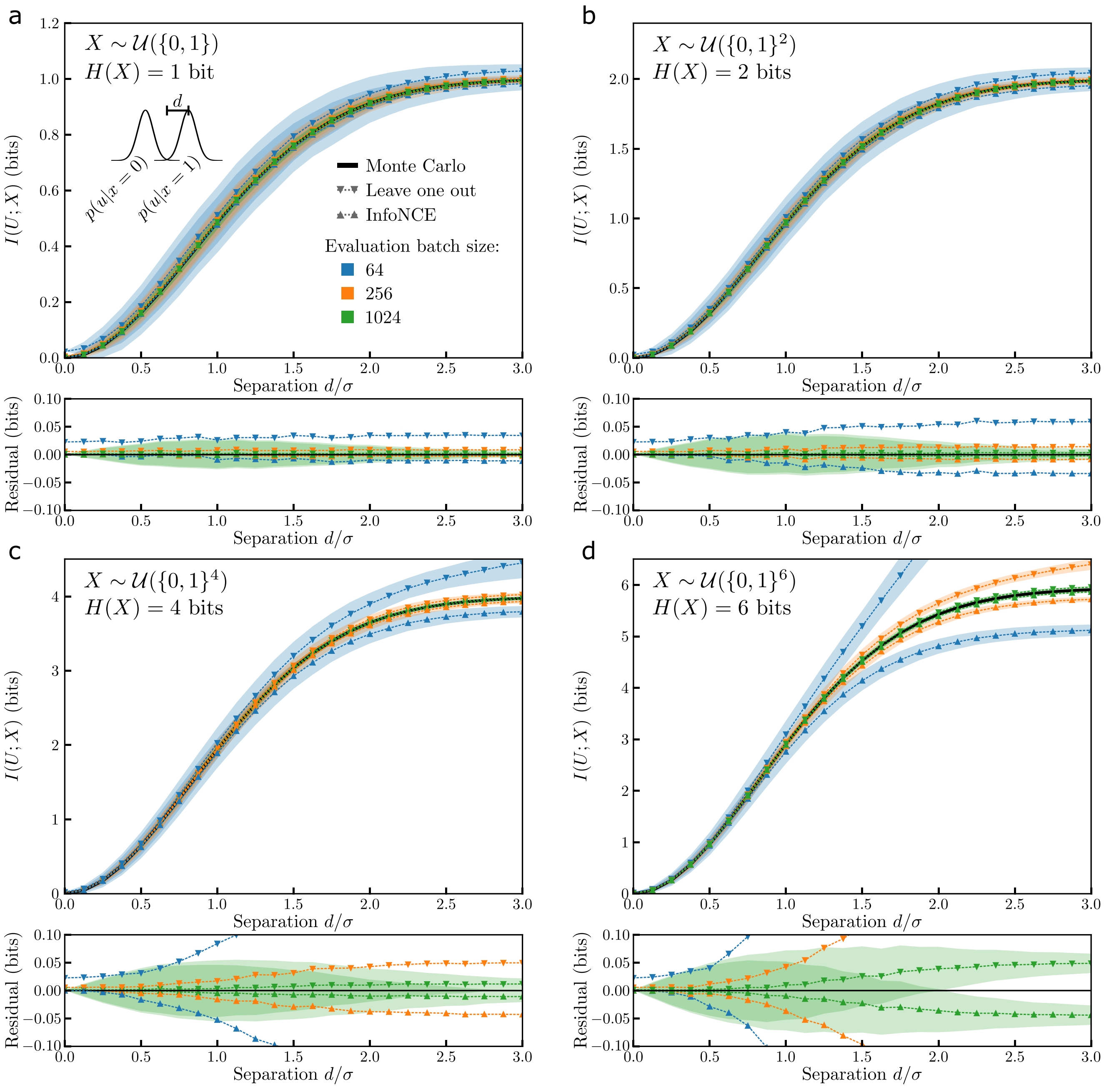

Fig. S4. Performance of mutual information bounds. A parameterized compression scheme encodes a discrete X to a separate normal distribution per outcome x, each with unit variance in $\mathbb{R}^{32}$ and spaced from the origin by a separation distance d. The expectation and standard deviation of the “leave one out” upper and InfoNCE lower bounds are the dashed line and shaded regions, respectively, evaluated over 4096 batches. In the residual plots, which show the difference between the bounds and the Monte Carlo estimate, only the standard deviations for the evaluation batch size of 1024 are displayed. Variables X with **(a)** one, **(b)** two, **(c)** four, and **(d)** six bits of entropy are used to generate the dataset for evaluation.

SI Dataset S1 (glass_data.tar.gz)

The train and validation splits of the glass data, consisting of local neighborhoods immediately preceding rearrangement events, derived from Ref. (13), have been attached to the manuscript as a directory of `.csv` files. Additionally, the same dataset was deposited on Figshare as a directory of `numpy` arrays (https://doi.org/10.6084/m9.figshare.24585150.v1)(26). The neighborhoods were subsequently "measured" as radial densities (Figs. 2 and 3 of the main text) or as per-particle descriptors (Fig. 4 of the main text); the code to process the neighborhoods is in the project Github repository. Also included in the dataset are the computed radial distribution functions, $g_{\mathrm{AA}}(r)$ and $g_{\mathrm{AB}}(r)$, for each quench.

References

1. SM Lundberg, SI Lee, A unified approach to interpreting model predictions in *Advances in Neural Information Processing Systems 30*, eds. I Guyon, et al. (Curran Associates, Inc.), pp. 4765–4774 (2017).
2. I Covert, SM Lundberg, SI Lee, Understanding global feature contributions with additive importance measures. *Adv. Neural Inf. Process. Syst.* **33**, 17212–17223 (2020).
3. C Molnar, *Interpretable Machine Learning: A Guide for Making Black Box Models Explainable.* (2022).
4. KA Murphy, DS Bassett, Interpretability with full complexity by constraining feature information in *International Conference on Learning Representations (ICLR).* (2023).
5. N Tishby, FC Pereira, W Bialek, The information bottleneck method. *arXiv preprint physics/0004057* (2000).
6. AA Alemi, I Fischer, JV Dillon, K Murphy, Deep variational information bottleneck. *Int. Conf. on Learn. Represent. (ICLR)* (2017).
7. A Kolchinsky, BD Tracey, S Van Kuyk, Caveats for information bottleneck in deterministic scenarios. *Int. Conf. on Learn. Represent. (ICLR)* (2019).
8. SS Schoenholz, ED Cubuk, DM Sussman, E Kaxiras, AJ Liu, A structural approach to relaxation in glassy liquids. *Nat. Phys.* **12**, 469–471 (2016).
9. D McAllester, K Stratos, Formal limitations on the measurement of mutual information in *International Conference on Artificial Intelligence and Statistics.* (PMLR), pp. 875–884 (2020).
10. AM Saxe, et al., On the information bottleneck theory of deep learning. *J. Stat. Mech. Theory Exp.* **2019**, 124020 (2019).
11. IE Aguerri, A Zaidi, Distributed variational representation learning. *IEEE Transactions on Pattern Analysis Mach. Intell.* **43**, 120–138 (2021).
12. B Poole, S Ozair, A Van Den Oord, A Alemi, G Tucker, On variational bounds of mutual information in *International Conference on Machine Learning.* (PMLR), pp. 5171–5180 (2019).
13. D Richard, et al., Predicting plasticity in disordered solids from structural indicators. *Phys. Rev. Mater.* **4**, 113609 (2020).
14. A Barbot, et al., Local yield stress statistics in model amorphous solids. *Phys. Rev. E* **97**, 033001 (2018).
15. M Tancik, et al., Fourier features let networks learn high frequency functions in low dimensional domains. *Adv. Neural Inf. Process. Syst.* **33**, 7537–7547 (2020).
16. J Lee, et al., Set transformer: A framework for attention-based permutation-invariant neural networks in *International conference on machine learning.* (PMLR), pp. 3744–3753 (2019).
17. D Maliniak, R Powers, BF Walter, The gender citation gap in international relations. *Int. Organ.* **67**, 889–922 (2013).
18. N Caplar, S Tacchella, S Birrer, Quantitative evaluation of gender bias in astronomical publications from citation counts. *Nat. Astron.* **1**, 1–5 (2017).
19. P Chakravartty, R Kuo, V Grubbs, C McIlwain, #CommunicationSoWhite. *J. Commun.* **68**, 254–266 (2018).
20. ML Dion, JL Sumner, SM Mitchell, Gendered citation patterns across political science and social science methodology fields. *Polit. Analysis* **26**, 312–327 (2018).
21. JD Dworkin, et al., The extent and drivers of gender imbalance in neuroscience reference lists. *Nat. Neurosci.* **23**, 918–926 (2020).
22. P Zurn, DS Bassett, NC Rust, The citation diversity statement: a practice of transparency, a way of life. *Trends Cogn. Sci.* **24**, 669–672 (2020).
23. J Dworkin, P Zurn, DS Bassett, (In)citing action to realize an equitable future. *Neuron* **106**, 890–894 (2020).
24. D Zhou, et al., Gender diversity statement and code notebook v1. 0. *Zenodo* (2020).
25. Z Budrikis, Growing citation gender gap. *Nat. Rev. Phys.* **2**, 346–346 (2020).
26. KA Murphy, DS Bassett, Regions of simulated glasses for binary classification about imminent rearrangement https://doi.org/10.6084/m9.figshare.24585150.v1 (2023).
